# Adaptation Under Pressure: Resistance and Stress Response Interplay in Clinical *Aspergillus fumigatus* Isolates

**DOI:** 10.3390/jof11060428

**Published:** 2025-06-02

**Authors:** Ivana Segéňová, Ján Víglaš, Tomáš Pagáč, Petra Olejníková

**Affiliations:** Institute of Biochemistry and Microbiology, Faculty of Chemical and Food Technology, Slovak University of Technology in Bratislava, 812 37 Bratislava, Slovakia; jan.viglas@stuba.sk (J.V.); tomas.pagac@stuba.sk (T.P.)

**Keywords:** *Aspergillus fumigatus*, resistance, azoles, persistence, tolerance, stress response, fitness cost, virulence

## Abstract

Understanding the interplay between antifungal resistance, stress adaptation, and virulence in *Aspergillus fumigatus* is critical for more effective treatment outcomes. In this study, we investigate six clinical isolates of *A. fumigatus* from the hospitals of the Czech Republic, focusing on their resistance profiles, stress responses, and survival mechanisms under antifungal pressure. Notably, we have shown that azole-susceptible strains were able to form persister cells under supra-MIC concentrations, highlighting an emerging non-genetic survival mechanism. Stress response profiling demonstrated differential susceptibility to agents targeting signal transduction pathways, as principal component analysis proved that even azole-resistant strains might rely on these pathways. Combinatorial treatment with posaconazole and dithiothreitol enhanced antifungal efficacy regardless of the susceptibility of the strains. Fitness assays revealed that azole resistance imposed a competitive disadvantage in azole-free conditions. In vivo virulence assessment in *Galleria mellonella* larvae revealed strain-specific pathogenicity that did not directly correlate with resistance. Together, our findings illustrate the multifactorial nature of fungal survival and emphasize that stress adaptation, tolerance, and persistence significantly affect treatment efficacy and outcomes, even in the absence of classical resistance mechanisms. Targeting stress response pathways emerges as a promising strategy to enhance the efficacy of existing antifungal agents and manage resistance in *A. fumigatus*.

## 1. Introduction

*Aspergillus fumigatus* is an ascomycete filamentous fungus commonly found in the environment, where it plays a crucial role in decomposing organic matter and contributing to the natural carbon cycle [1,2,3]. However, it is a significant opportunistic pathogen, particularly affecting immunocompromised individuals [4]. Due to its ability to produce airborne conidia (2–3 µm), *A. fumigatus* can easily enter the respiratory tract of the host, where, under normal immune function, spores are effectively cleared through the innate immune response involving alveolar macrophages and neutrophils [5]. In contrast, in patients undergoing immunosuppressive therapy or suffering from severe underlying disease, inhaled spores can lead to various infections collectively termed aspergilloses [2,6,7]. Among these, invasive aspergillosis is the most severe form, primarily affecting the lungs, and in some cases, disseminating to other organs such as the central nervous system [8,9]. Globally, invasive fungal infections contribute to more than 1.5 million deaths annually, with *A. fumigatus* being the leading cause of mortality in aspergillosis, exhibiting fatality rates exceeding 50% even with appropriate treatment [7,10,11]. The increasing incidence of invasive aspergillosis, coupled with the emergence of antifungal-resistant strains, poses a significant challenge to clinical management and highlights the need for improved strategies.

The primary therapeutic options for aspergillosis include azoles, polyenes, and echinocandins [5]. Azoles, such as voriconazole, itraconazole, posaconazole, and isavuconazole, are the preferred first-line treatments due to their efficacy, tolerability, and oral bioavailability [2,12]. These compounds exert their antifungal activity by inhibiting ergosterol biosynthesis, a vital component of the fungal cell membrane necessary for membrane fluidity and integrity [13]. However, increasing azole resistance in *A. fumigatus* poses a significant challenge in clinical settings, with resistance mechanisms including spontaneous mutations in the *cyp51A* gene and tandem repeat (TR) mutations that enhance gene transcription [3,14]. Notably, approximately 50% of azole-resistant *A. fumigatus* strains lack *cyp51A* mutations, suggesting alternative resistance mechanisms such as upregulation of ABC and MFS transporters, gain-of-function mutations in transcription factors, and alterations in sterol biosynthesis pathways [2,15].

Beyond resistance, emerging evidence indicates that *A. fumigatus* can survive antifungal therapy through two additional phenomena: tolerance and persistence. Tolerance refers to the ability of the entire fungal population to survive at drug concentrations above the minimal inhibitory concentration (MIC), while persistence is characterized by a small subpopulation (<1%) that can survive prolonged antifungal exposure without genetic mutations conferring resistance [7,16]. These mechanisms may contribute to treatment failure even in azole-susceptible strains, potentially leading to chronic infections and an increased likelihood of developing resistance due to prolonged fungal survival under selective pressure [10,17].

In this study, we investigate resistance, tolerance, and persistence in clinical *Aspergillus fumigatus* isolates, some of which were obtained from immunocompromised patients during the early stages of the COVID-19 pandemic in the Czech Republic. One environmental isolate (*A. fumigatus* CCM F-386, producer of helvolic acid) was included in our experimental setup. This strain provided additional insight into antifungal resistance mechanisms beyond those found in clinical isolates. Using susceptibility testing, we analysed azole resistance linked to *cyp51A* mutations and alternative pathways. We examined the correlation between resistance and fitness cost in mixed populations, as well as between resistance and virulence using the *Galleria mellonella* in vivo model. Additionally, we evaluated persister cell formation. Our findings provide valuable insight into the adaptive strategies of *A. fumigatus* and their implications for antifungal therapy. The results highlight the need for a comprehensive approach to understanding and managing fungal resistance beyond conventional susceptibility testing, incorporating persistence and tolerance studies to improve clinical outcomes.

## 2. Materials and Methods

### 2.1. Aspergillus fumigatus Strains

This study analysed six clinical isolates of *A. fumigatus* from the Czech Republic (Table 1). Clinical strains were obtained from patients with varying health conditions, including COVID-19, acute suppurative otitis, and cough. The strain selection was based on documented clinical treatment failure and genetic variability of isolates, ensuring a representative set for resistance analysis. We selected representatives from the group with confirmed mutation in the *cyp51* gene (i.e., Af6367, Af6600, Af6601) and from the group without confirmed mutation in the *cyp51* gene (i.e., Af6651, Af6658, Af3384). Confirmed mutations in the *cyp51* gene included L98H or TR46/Y121F/T289A. The environmental strain Af386 was obtained from the Czech collection of microorganisms and was included in our experimental settings as well (Česká sbírka mikroorganismů, Masarykova univerzita, Brno).

### 2.2. Antifungal Susceptibility Testing

#### 2.2.1. Agar Screening Method for Azole Resistance

RPMI 1640 medium, supplemented with 2% glucose and buffered with MOPS, was prepared following EUCAST recommendations specified in E. Def 10.2 manual. Agar plates were supplemented with voriconazole (VRC, 2 mg/L), itraconazole (ITC, 4 mg/L), and posaconazole (PSC, 0.5 mg/L) selectively to allow the growth of resistant strains while inhibiting wild-type populations. Instead of standard 4-well plates, 5 cm Petri dishes were used.

Conidial suspensions were obtained using 0.1% Tween-80 (Biolife, Italy) and adjusted to 3.0 × 10^5^ conidia/mL. To avoid excess liquid spread, 5 µL of conidial suspension was applied onto a sterile 5 mm paper disc placed at the centre of the solidified medium.

Plates were incubated at 37 °C in the dark for 48 h, after which fungal growth was assessed. Strains exhibiting growth only on control (azole-free) plates were classified as susceptible, whereas those showing growth on azole-containing plates were considered as resistant [18].

#### 2.2.2. MIC Determination

The minimum inhibitory concentration (MIC) values of azoles (voriconazole—VRC, itraconazole—ITC, posaconazole—PSC), polyene (amphotericin B—AMB), and echinocandins (caspofungin—CAS, micafungin—MIK, anidulafungin—ANI) (Sigma-Aldrich, St. Louis, MI, USA) were determined using the agar dilution method. These antifungals were dissolved in dimethyl sulfoxide (DMSO) and diluted (Table S1).

Petri dishes (5 cm diameter) were filled with 50 µL of diluted antifungal solution and 5 mL of RPMI 1640 medium, ensuring the final DMSO concentration never exceeded 1% *v*/*v*. Conidial suspensions were prepared as described in Section 2.2.1, and inoculation was performed accordingly.

Plates were incubated at 30 °C in the dark until control colonies reached the edge of the plate. MIC values were determined by comparing fungal growth (colony diameter) to that of untreated controls. Each experiment was repeated at least three times [18,19].

### 2.3. Susceptibility to Stress-Inducing Agents and Adaptive Response Modulators

To evaluate fungal response to different stress conditions, potato dextrose agar (PDA) (Merck, Germany) was used as the nutrient medium to ensure optimal growth conditions. Various chemical agents, including menadione, rapamycin, FK506, cyclosporine A, brefeldin A, 4-nitroquinoline-1-oxide (4 NQO), tunicamycin, and benomyl (Sigma Aldrich, Saint Louis, Missouri, USA), were dissolved in DMSO and diluted to the required concentrations (Table S1).

Other agents, such as hydrogen peroxide, dithiothreitol (DTT), and sodium dodecyl sulfate (SDS) (Sigma Aldrich, USA), were diluted in sterile distilled water.

Sodium chloride (NaCl), lithium chloride (LiCl), and Congo red (Erba Lachema, Czech Republic) were added to the medium before sterilization.

Petri dishes (5 cm diameter) were filled with 50 µL of prepared stress-inducing agent solution and 5 mL of PDA, ensuring the final DMSO concentration never exceeded 1% *v*/*v*. Inoculation and incubation procedures followed the methodology described in Section 2.2.1.

The MIC80 value (the concentration at which 80% of fungal growth is inhibited compared to the control) was determined for each condition. Control colonies were grown on DMSO-supplemented and non-supplemented media. Each test was repeated at least three times.

### 2.4. The Impact of Additional Stress-Inducing Agents on the Antifungal Efficacy

To evaluate potential improvements in the efficiency of azoles, 2-fold concentrated solutions of each tested compound (DTT, tunicamycin, brefeldin A, rapamycin, FK506, cyclosporine A) and each azole (VRC, ITC, PSC) were prepared (Table S2). Azole solution (25 µL) and stress-inducing agent (25 µL) were pipetted into sterile 5-cm Petri dishes, followed by the addition of 5 mL of PDA.

To compare individual effects, media containing 50 µL of either azole or the tested compound alone were also prepared. Fresh conidial suspension (5 µL of 3.0 × 10^5^ conidia/mL) was inoculated on a 5 mm sterile paper disc placed at the centre of each plate. Control colonies were grown on DMSO-supplemented and non-supplemented media. Cultivation was conducted at 30 °C in the dark until control colonies reached the edge of the plate. Changes in the effectiveness of azoles were assessed by comparing fungal growth in the presence of both compounds versus each compound individually. Experiments were performed in two biological replicates.

### 2.5. Persister Formation

To determine the presence of persister cells, 10 cm Petri dishes were filled with inoculated (10^3^ conidia/mL) RPMI 1640 growth media supplemented with 2% glucose and buffered with MOPS. After solidification, a sterile 1 × 8 cm paper strip was placed in the centre of each plate.

Azole solutions (VRC, ITC, and PSC) were prepared at supra-MIC concentrations (Table S3), and 100 µL of each solution was applied evenly to the paper strip. Plates were incubated at 37 °C in the dark for four days, with daily monitoring. Persisters were defined as isolated colonies appearing within the inhibition zone [7].

### 2.6. Fitness Comparison of Azole-Resistant and Azole-Susceptible Strains

To assess fitness differences, we used a protocol devised by Chen et al. (2024) [20]. Briefly, conidial suspensions of resistant and susceptible strains were prepared at 10^5^ conidia/mL and mixed at a 1:1 ratio (final concentration 2 × 10^5^ conidia/mL). The mixture was serially diluted (10^−5^ to 10^−8^), and 1 mL of each dilution was evenly spread onto PDA plates. Co-cultivation was performed on (*i*) azole-free PDA medium and (*ii*) voriconazole (VRC) containing PDA plates. A VRC concentration of 1 mg/L, if co-cultured with Af6600, or 0.5 mg/L, if co-cultured with Af6367, was tested (concentrations are ≥MIC for VRC-susceptible strains, but allow VRC-resistant strains to grow, Figure S1).

After 48 h at 37 °C, colony counts on azole-supplemented and non-supplemented media were compared. Conidia from non-diluted cultures grown on azole-free medium were harvested and transferred every 48 h for five passages [20]. The fitness of strains was compared three times.

### 2.7. Virulence Comparison Using the Galleria mellonella In Vivo Model

*Galleria mellonella* larvae that had reached the final stage of larval development were used for testing. Prior to the experiment, larvae were incubated in the water bath (57 °C, 9 s) to avoid the formation of silk for three weeks and stored at 15 °C in dark conditions until the experimental work. *A. fumigatus* strains were cultivated on PDA medium at 30 °C, and cultures ≤ 7 days old were used. The conidial suspension was prepared in PBS supplemented with 0.01% Tween-80 and adjusted to a concentration of 1 × 10^7^ conidia/mL. Healthy non-melanised larvae (250–300 mg) were randomly selected for the experiment and divided into nine groups (10 larvae/group): seven groups were infected with particular *A. fumigatus* strains, larvae injected with PBS + 0.01% Tween-80, and intact larvae served as controls. Inoculum (10 µL/larva) was injected via the last proleg (1 × 10^5^ conidia/larva) using a Hamilton syringe. Larvae were incubated at 37 °C under dark conditions, and survival was monitored daily for nine days. Larvae were considered dead if they did not respond to touch stimuli.

#### *Galleria mellonella* Larvae Origin

Larvae used for the experiments are bred in our laboratory. Briefly, *Galleria mellonella* moths are kept in a controlled environment at laboratory temperature in conditions with natural cycles of sunshine. After the eggs are laid on filter paper, they are placed in a Petri dish filled with specially prepared food (a mixture of wheat flour, cornmeal, wheat bran, honey, dried whole milk, beeswax, dried yeast, and glycerol). After hatching and during growth, the larvae are regularly fed and kept at laboratory temperature. After reaching the last instar (200–300 mg), the enzyme responsible for silk production is denatured by bathing at 57 °C for 9 s (empirically optimized conditions) to prevent cocoon formation and transformation of larvae into moths. Following enzyme inactivation, larvae are stored in a thermostat at 15 °C.

### 2.8. Statistical Analysis

Principal component analysis (PCA) was performed using the mean of corresponding MIC values across all tested stress-inducing agents. The analysis was conducted in Python 3.10 using the scikit-learn library (v1.4.1) for dimensionality reduction and seaborn (v0.13.2) for visualization. MIC data were centred and scaled using z-score normalization prior to analysis.

The Student’s *t*-test was used to evaluate statistically significant differences in MIC values between azole-resistant and azole-susceptible strains for each individual stressor. One-way ANOVA was applied when comparing susceptibility to multiple agents associated with a common stress type—such as oxidative stress (H_2_O_2_ and menadione), cell wall/membrane stress (Congo red and SDS), inhibition of signaling pathways (cyclosporine, rapamycin, FK506), or endoplasmic reticulum stress (DTT, brefeldin A, tunicamycin). Both Student’s *t*-test and one-way ANOVA analyses were performed using OriginPro 6.0.

To assess group-level differences across multiple dependent variables simultaneously, multivariate analysis of variance (MANOVA) was conducted in Python using the statsmodels library (v0.14.1). This allowed the detection of overall susceptibility pattern differences between resistant and susceptible strains. For example, MANOVA applied to oxidative stressors (H_2_O_2_ and menadione) and cell wall stressors (SDS and Congo red) did not reveal statistically significant differences between the two groups (Wilks’ λ > 0.8, *p* > 0.05 in both cases).

Regarding the impact of additional stress-inducing agents on the antifungal efficacy, statistical significance across the treatment conditions was evaluated using one-way ANOVA (OriginPro 6.0). Where significant, pairwise comparisons were performed using paired Student’s *t*-tests with Bonferroni correction (* 0.05 > *p* > 0.01, ** 0.01 > *p* > 0.001, and *** 0.001 > *p*).

## 3. Results

### 3.1. Antifungal Susceptibility Determination In Vitro

**Figure 1:**
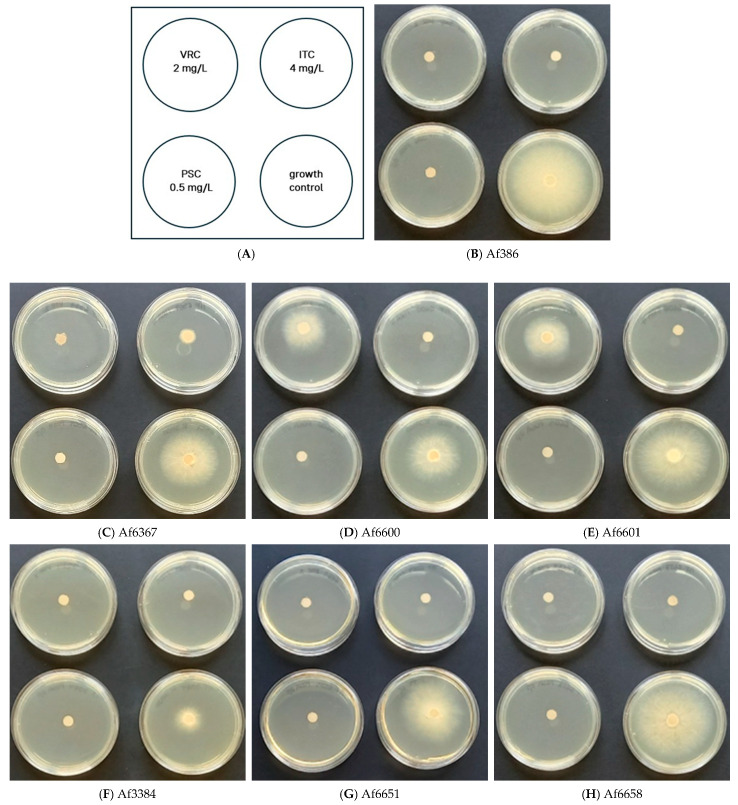
Assessment of *A. fumigatus* resistance using azole-containing agar plates. (**A**) Schematic representation of the assay layout, including test conditions for voriconazole (VRC), itraconazole (ITC), and posaconazole (PSC), alongside a control plate without azoles. (**B**–**H**) Growth patterns of *A. fumigatus* strains after 48 h of incubation on azole-supplemented and control media. Strains Af6600 and Af6601 exhibited high resistance to voriconazole, while strain Af6367 showed reduced susceptibility to both voriconazole and itraconazole. In contrast, strains Af386, Af3384, Af6651, and Af6658 were classified as azole-susceptible.

The susceptibility or resistance of *A. fumigatus* isolates to clinically relevant antifungal agents was assessed using the EUCAST-recommended protocol [18] (Figure 1A–H). Based on the results, four strains (Af386, Af3384, Af6651, and Af6658) were classified as susceptible to azoles. In contrast, strains Af6600 and Af6601, carrying the TR46/Y121F/T289A mutation, exhibited marked growth in the presence of voriconazole, comparable to the control, confirming their high-level resistance.

Strain Af6367, which harbours the L98H mutation in *cyp51A* but lacks a tandem repeat in the promoter region, displayed significantly reduced but still detectable growth in the presence of voriconazole and itraconazole. This suggests a moderate resistance phenotype, distinct from the TR46/Y121F/T289A strains.

To quantify resistance levels and to confirm the observed growth patterns, we determined MIC values for all strains using media containing varying azole concentrations (Table S1). The results (Table 2 and Figure S1) were compared to EUCAST clinical breakpoints (CBs) [21], which integrate microbiological data, pharmacokinetics, and clinical outcomes [10].

The TR46/Y121F/T289A-carrying strains (Af6600 and Af6601) exhibited high MIC values for voriconazole, a characteristic resistance profile associated with this type of mutation [22]. Even at the highest tested voriconazole concentration (10 mg/L), growth inhibition reached only 50% and 70%, respectively (Figure S1), confirming substantial resistance. These strains also displayed elevated MICs for itraconazole, bordering the concentration used in the EUCAST screening test, further supporting a multi-azole resistance phenotype.

Strain Af6367, carrying the L98H mutation, exhibited high resistance to itraconazole and moderate resistance to voriconazole (Table 2). For itraconazole, a MIC70 value was determined, as the inhibition rate did not change with increasing azole concentration (Figure S1).

None of the tested strains exhibited resistance to posaconazole. However, strains Af6367 and Af6600 exhibited the MIC value at the EUCAST cutoff for ATU (Areas of Technical Uncertainty). EUCAST guidelines indicate that reduced susceptibility to itraconazole can predict posaconazole resistance, yet our findings suggest these strains should be classified as having reduced susceptibility rather than resistance to posaconazole [21].

Given the clinical relevance of amphotericin B (AMB) and echinocandins in aspergillosis treatment, we extended susceptibility testing to these antifungals. Surprisingly, six out of seven strains exhibited high MIC values for AMB, an unusual finding given that resistance to AMB in *A. fumigatus* is rare (~2% prevalence) [23]. This suggests a potential adaptive resistance mechanism, warranting further investigation.

As echinocandins exert a fungistatic effect on *A. fumigatus*, we determined MIC80 values (the concentration inhibiting 80% of the fungal population). By comparing our data with previous studies [24,25,26], we observed a similar MIC range, suggesting no significant reduction in echinocandin susceptibility across the tested strains.

### 3.2. Persistence

In clinical practice, treatment failure has been observed even in strains classified as susceptible under laboratory conditions. This phenomenon is associated with the formation of persister cells in the presence of an antifungal compound during treatment. In fungal cells, persistence is defined as the ability of a small subpopulation of cells (<1%) within an isogenic population to survive and grow at drug concentrations ≥ 8× the MIC [17].

**Figure 2:**
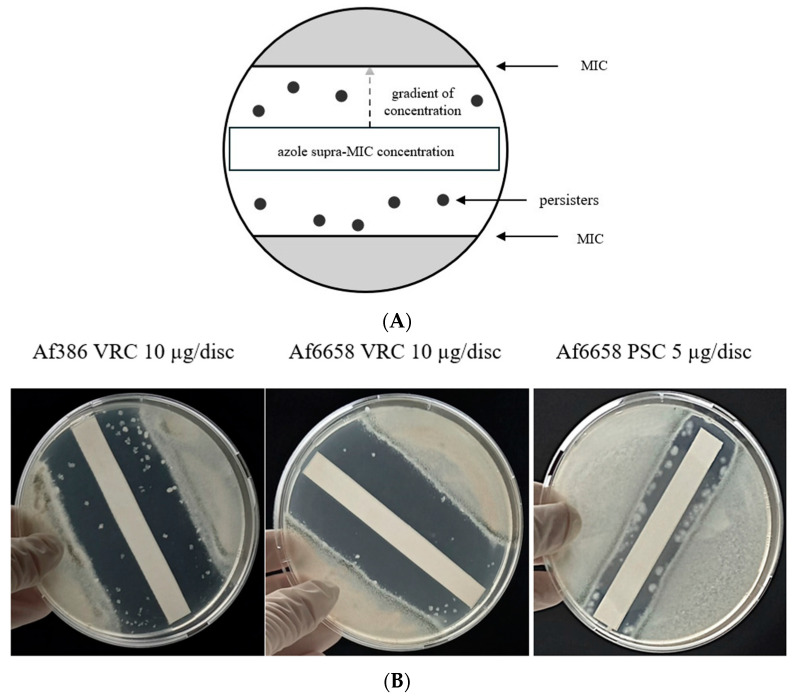
Detection of persister cell formation in *A. fumigatus*. (**A**) Schematic representation of the persistence assay. Colonies that grow within the inhibition zone are classified as persisters. (**B**) Detection of persister cells in *A. fumigatus* strains Af386 and Af6658 in the presence of voriconazole (VRC, 10 µg/disc). Additionally, strain Af6658 exhibited persister growth in the presence of posaconazole (PSC, 5 µg/disc).

For this analysis, we tested three clinically relevant azoles (VRC, ITC, PSC) at supra-MIC concentrations. During cultivation, inhibition zones formed around the azole-containing discs, reflecting the susceptibility profiles of individual strains (Figure 2A). Within these zones, persister cells appeared as isolated single colonies, distinct from the main fungal population.

Persister growth was observed in three azole-susceptible strains (Table S3), particularly under high VRC concentrations exceeding their MIC values. Additionally, strain Af6658 exhibited persister growth in the presence of PSC at similarly elevated concentrations (Figure 2B).

The number of persisters remained relatively stable throughout the cultivation period, with only slight fluctuations. Notably, strain Af3384 produced very faint colonies within the inhibition zone. However, microscopic analysis confirmed conidial germination and hyphal growth, indicating persistence.

In contrast, azole-resistant strains exhibited significantly smaller inhibition zones, and no persister formation was observed (Table S3).

### 3.3. Susceptibility of A. fumigatus to Stress-Inducing Agents and Adaptive Response Modulators

To better understand the stress response mechanisms of *A. fumigatus*, we evaluated the susceptibility of clinical and environmental isolates to a panel of stress-inducing agents that target key cellular processes. These included compounds affecting cell wall integrity, osmotic balance, oxidative stress response, signal transduction pathways, endoplasmic reticulum (ER) function, and cytoskeletal stability. This comprehensive approach enabled a comparative analysis of how resistance mechanisms influence overall stress tolerance and, conversely, how stress adaptation might impact antifungal susceptibility. The overview of the results is presented in Table 3.

Considering the conserved signaling pathways that are important for adaptive response activation, we evaluated the susceptibility of these strains to their inhibitors. Since these molecules do not fully inhibit fungal growth (100% inhibition), various MIC40-MIC80 values were evaluated to reflect strain-specific properties. Differences in susceptibility to rapamycin, FK506, and cyclosporine A suggest variations in TOR kinase and calcineurin pathway regulation. Strain Af3384 was the least susceptible to rapamycin, with only 40% growth inhibition (MIC40 value), whereas the remaining strains exhibited up to 60% inhibition (MIC60 values, Figure S3). Strains Af6600 and Af6651 displayed 10-fold higher MIC60 values compared to other azole-resistant or susceptible strains. For FK506 and cyclosporine A, strain Af3384 exhibited the highest MIC80 value among the tested strains. Overall, strain Af3384 was the most tolerant.

**Figure 3:**
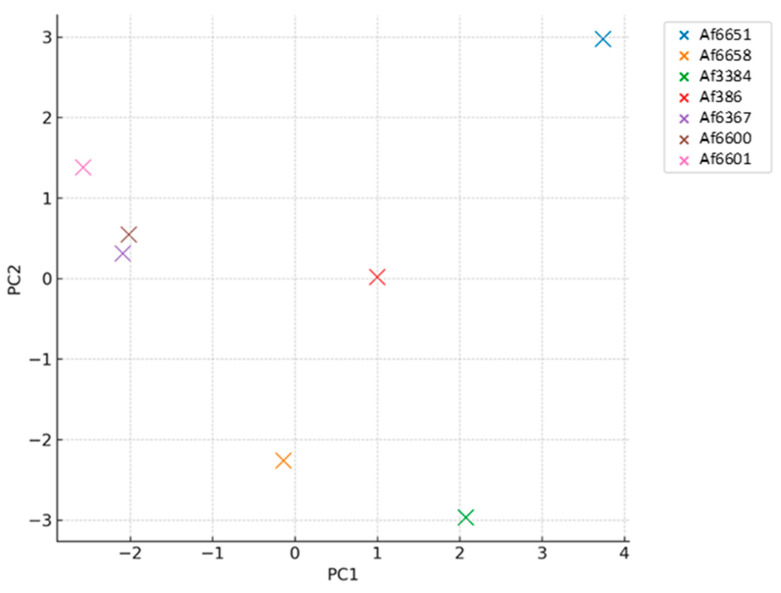
Principal component analysis (PCA) of stress susceptibility in *A. fumigatus* strains. PCA was based on z-score normalized MIC values. The figure was generated in Python using the scikit-learn and seaborn libraries.

To explore broader patterns, we performed principal component analysis (PCA) based on corresponding MIC values for all tested compounds (Figure 3). The PCA revealed a distinct clustering of azole-resistant clinical isolates (Af6367, Af6600, Af6601), indicating similar stress response profiles. In contrast, azole-susceptible strains (Af3384, Af6651, Af6658) were more dispersed within the PCA space, reflecting greater phenotypic variability. The environmental isolate (Af386) did not cluster with either clinical group and was therefore excluded from further statistical comparisons.

To determine differences in susceptibility between the two groups, we applied Student’s *t*-tests to MIC means for individual compounds (see Table 4). For pathways targeted by multiple agents (e.g., oxidative stress: H_2_O_2_ and menadione), one-way ANOVA was used to evaluate group-level differences.

Due to the uniformly high MIC80 values for benomyl in resistant strains—likely reflecting intrinsic resistance via β-tubulin mutations—this compound was excluded from statistical analyses. Based on the PCA-derived grouping, isolates were classified as either azole-resistant or azole-susceptible, and subsequent comparisons were conducted accordingly.

Azole-resistant isolates exhibited increased susceptibility to calcineurin inhibitors, including cyclosporine A (mean MIC80: 1.16 ± 0.69 vs. 6.23 ± 3.10) and FK506 (0.06 ± 0.02 vs. 0.27 ± 0.22), suggesting potential vulnerability to this pathway. These strains were also more susceptible to brefeldin A, an ER–Golgi trafficking inhibitor (9.56 ± 4.54 vs. 21.5 ± 14.7). In contrast, azole-susceptible strains showed greater susceptibility to SDS (0.02 ± 0.01 vs. 0.04 ± 0.01) and menadione (0.02 ± 0.01 vs. 0.07 ± 0.03). However, due to small absolute differences and low intra-group variability, results for SDS and menadione should be interpreted with caution.

Despite general group-level trends, substantial strain-level heterogeneity was observed. For example, SDS (a membrane-disrupting detergent) and Congo red (a chitin-binding dye affecting cell wall assembly by binding to β-1,3-glucans [27]) induced highly variable responses across individual strains. While SDS susceptibility was consistently higher in azole-susceptible strains, no clear pattern was observed for Congo red.

Oxidative stressors showed similarly divergent outcomes. Menadione, which generates superoxide radicals, elicited significantly higher MIC80 values in resistant strains, whereas hydrogen peroxide, mediating peroxide-induced damage, showed no significant differences between groups.

To assess whether these observations reflect broader pathway-specific differences, we applied MANOVA (v0.14.1) to grouped stressors (e.g., oxidative stress, ER stress). No statistically significant differences were found, indicating that stress tolerance does not correlate consistently with azole resistance status.

These findings underscore the complexity and plasticity of fungal stress adaptation. Rather than being strictly linked to azole resistance, stress phenotypes likely emerge from distinct evolutionary trajectories, shaped by diverse and possibly independent tolerance mechanisms.

Given the high degree of intra-group variability, we also analysed individual strain profiles to identify outlier MIC80 values. This approach revealed unique stress response signatures, which may reflect adaptive traits unrelated to azole resistance. These individual profiles emphasize the importance of considering strain-specific characteristics when evaluating antifungal susceptibility and predicting treatment outcomes.

Regarding cell wall integrity as a critical factor influencing azole susceptibility, Congo red serves as a useful indicator of fungal susceptibility level based on cell wall robustness. The results indicate that some strains (Af6600, Af3384, Af6651, and the environmental strain Af386) show higher tolerance to the presence of Congo red (MIC80 values in the range of 3.2–7.1 mM). It can be speculated that strains with higher MIC80 values may have enhanced cell wall integrity, as it plays a crucial role in adaptation to Congo red [27].

For osmotic stress adaptation, a key factor in conidial germination, growth, and virulence in *A. fumigatus* [28], the tested strains were exposed to NaCl and LiCl. Most strains displayed similar NaCl tolerance (~1.5–1.7 M), except strain Af6651 (0.95 ± 0.25 M), which showed increased susceptibility. In contrast, strain Af3384 had the highest tolerance to LiCl (107± 5.0 mM) among all strains.

To assess the role of efflux pumps in tolerance mechanisms, we tested *A. fumigatus* strains against 4-nitroquinoline-1-oxide (4 NQO), which, besides its DNA-damaging effect, serves as a substrate for the AtrF efflux pump, a key contributor to azole efflux in *A. fumigatus* [29]. Strain Af6367 exhibited the highest MIC80 values, suggesting increased basal activity of the AtrF efflux pump, which may contribute to azole resistance in conjunction with the L98H mutation in the *cyp51A* gene.

Overall, our results demonstrate that *A. fumigatus* stress responses are shaped by complex, strain-specific factors that do not consistently align with azole resistance status. While certain trends were observed, such as increased susceptibility of resistant strains to calcineurin and ER stress inhibitors (cyclosporine A, FK 506; brefeldin A), no uniform pattern emerged across stress pathways. The substantial phenotypic variability within both azole-resistant and azole-susceptible groups underscores the importance of considering individual strain behaviour rather than relying solely on resistance classification. These findings suggest that antifungal resistance and cellular stress adaptation likely follow independent evolutionary trajectories, and that exploring strain-specific stress vulnerabilities may offer complementary avenues for therapeutic intervention.

### 3.4. Interaction Between Antifungal Compounds and Signaling Pathway Modulators

Antifungal agents act by targeting specific cellular functions, often activating stress responses that can influence drug effectiveness [30]. Since the inhibition of key stress response pathways may enhance the action of antifungal agents, we examined the impact of signaling pathway modulators, i.e., endoplasmic reticulum stress-inducers (DTT, brefeldin A, tunicamycin) and TOR kinase and calcineurin pathway inhibitors (rapamycin, FK506, and cyclosporine A) on the susceptibility of *A. fumigatus* strains to azoles.

To evaluate the contribution of signalling pathways to stress adaptation, we selected compound concentrations that reduced fungal growth to 40–60% of the control levels (Table S2). By comparing fungal growth under single-agent versus combined conditions, we evaluated whether pathway modulation changed azole efficacy.

To investigate the effect of combined antifungal and stress-inducing agent exposure, we tested two *A. fumigatus* strains with distinct susceptibility profiles: Af6658—a clinical azole-susceptible strain, and Af6600—a clinical azole-resistant strain.

Among the tested azoles, posaconazole showed the highest antifungal activity, as demonstrated by the lowest MIC values (Table 2). This high efficacy is likely due to its stronger binding affinity for the target enzyme Cyp51A compared to other triazoles. This enhanced binding may also trigger a stronger activation of fungal stress responses, increasing reliance on pathways.

**Figure 4:**
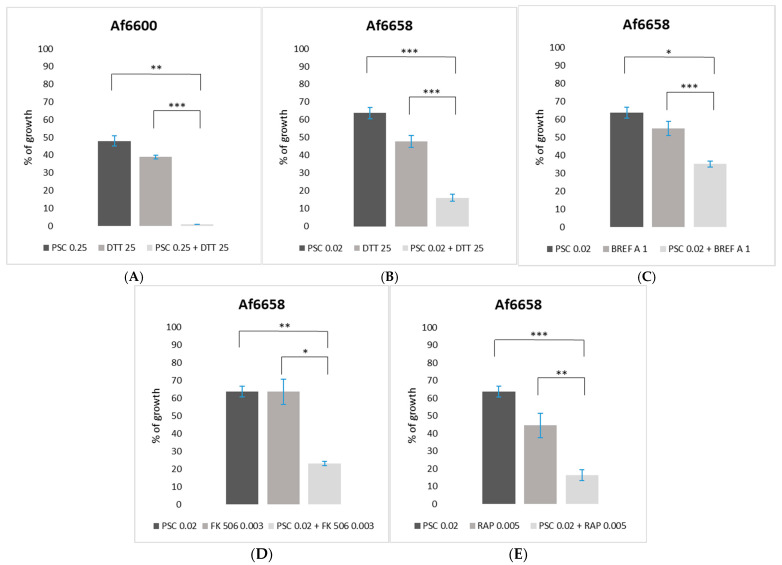
(**A**–**E**). Effect of azole and stress-inducing agent combinations on *A. fumigatus* growth. Comparison of fungal growth in the presence of individual azoles or stress-inducing agents versus their combined effect. Results where the combination of compounds resulted in at least a 20% reduction are shown. To determine statistical significance, pairwise comparisons were performed using paired Student’s *t*-tests with Bonferroni correction (* 0.05 > *p* > 0.01, ** 0.01 > *p* > 0.001, and *** 0.001 > *p*). Abbreviations: PSC-posaconazole, BREF A-brefeldin A, RAP-rapamycin, FK 506 (mg/L), DTT-dithiothreitol (mM).

The most notable increase in susceptibility occurred exactly when a signaling pathway modulator was combined with posaconazole. Strain Af6658 exhibited the broadest increase in sensitivity, with growth reduced by approximately 20% when posaconazole was combined with brefeldin A (Figure 4C). A variation in the degree of inhibitory effect was also observed in the case when posaconazole was combined with FK506, rapamycin, or DTT (Figure 4D,E,B). Despite being azole-susceptible, Af6685 appeared particularly vulnerable to modulators of TOR and ER-Golgi signaling (FK506, rapamycin, brefeldin A), suggesting these pathways play a critical compensatory role in this strain under azole stress. In contrast, in the azole-resistant strain Af6600 harbouring the TR46/Y121F/T289A mutation, the combination of posaconazole with DTT led to complete growth inhibition (Figure 4A).

These findings highlight the potential for targeting specific stress response pathways to enhance azole efficacy, particularly in a strain-specific manner.

### 3.5. The Correlation Between Resistance and Fitness Cost of A. fumigatus Strains

Antifungal resistance is often associated with a fitness cost, which can place resistant strains at a competitive disadvantage in natural environments. To evaluate this effect, we conducted in vitro co-culture experiments to assess the relative fitness of azole-resistant and azole-susceptible strains.

For this analysis, we selected: (*i*) voriconazole-resistant strains carrying distinct *cyp51A* mutations: strain Af6600 (TR46/Y121F/T289A) and strain Af6367 (L98H point mutation), (*ii*) a voriconazole-susceptible clinical isolate (Af6658), and (*iii*) a voriconazole-susceptible environmental strain (Af386).

**Figure 5:**
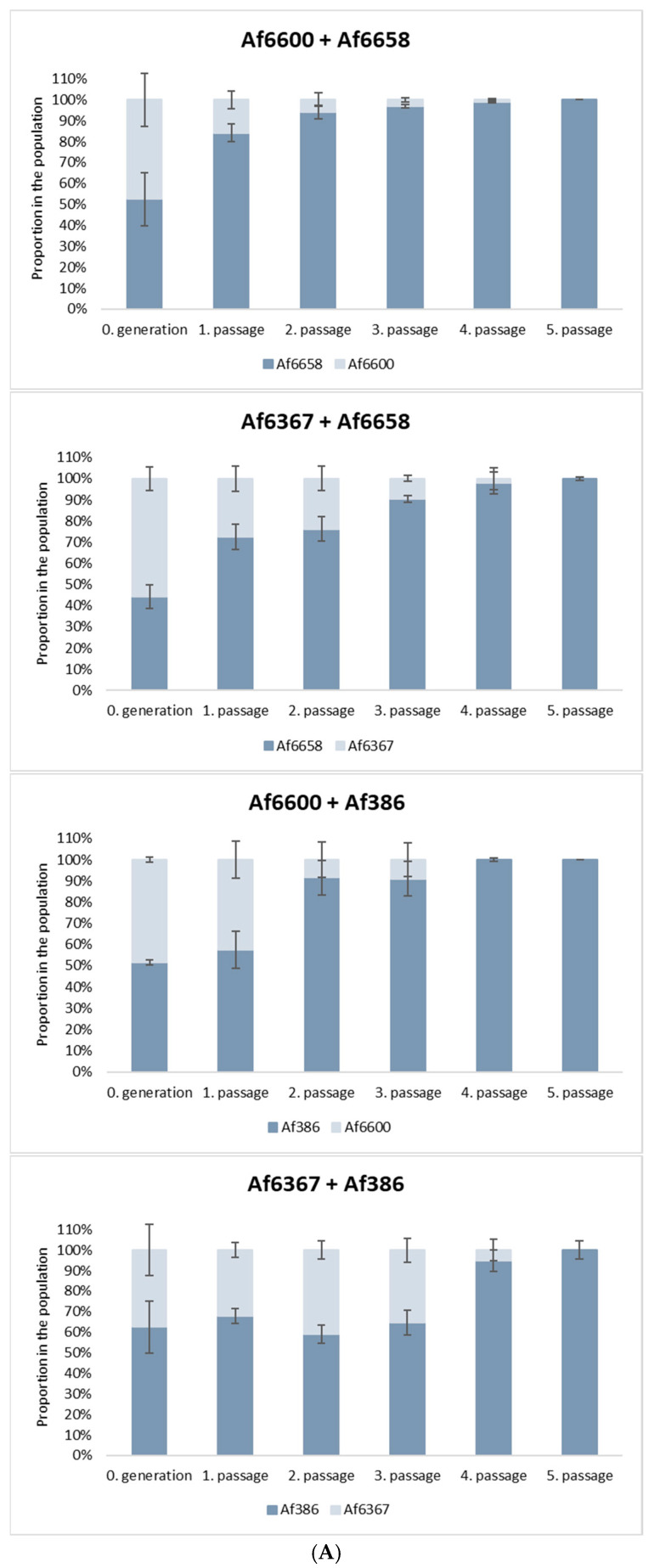

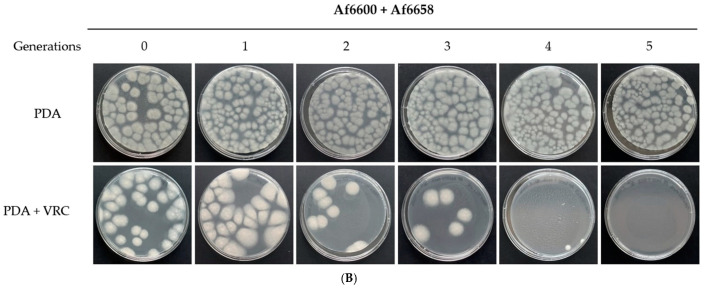
In vitro fitness assay of azole-resistant and azole-susceptible *A. fumigatus* strains. (**A**) Proportion (%) of azole-resistant and azole-susceptible isolates over five generations (representing a 10^−6^ dilution after harvesting conidia from plates after co-cultivation, inoculum for co-cultivation was 2 × 10^5^ conidia/mL). Each passage was performed after 48 h of cultivation. To distinguish between resistant and susceptible strains, voriconazole was applied at 1 mg/L for Af6600 and 0.5 mg/L for Af6367. Susceptible strains are represented in dark blue, and resistant strains in light blue. (**B**) Competitive fitness of azole-resistant (Af6600) and susceptible (Af6658) strains. Changes in colony proportions were monitored on PDA agar plates supplemented or non-supplemented with voriconazole. A significant decline in the abundance of the resistant isolate was observed on azole-containing medium over successive passages (representing a 10^−6^ dilution after harvesting conidia from plates after co-cultivation, inoculum for co-cultivation was 2 × 10^5^ conidia/mL).

As shown in Figure 5A, azole-susceptible strains progressively outcompeted resistant strains with each successive passage in co-culture. Notably, strain Af6658 exhibited a 30% increase in relative abundance after the first passage when competing with both resistant strains. For strain Af386, a 35% increase was observed after the second passage, particularly in competition with strain Af6600. The graphs illustrate the results obtained from the 10^−6^ dilution, with similar patterns observed across the other dilutions (10^−5^, 10^−7^, 10^−8^).

In co-culture with the strain Af6367, the relative proportions of both strains remained stable across passages, although the susceptible strain ultimately prevailed. By the fourth or fifth passage, the proportion of azole-resistant strains had declined to ≤5% in all experimental conditions.

These findings indicate that azole resistance imposes a significant fitness cost, reducing the competitive advantage of resistant isolates in the absence of selective drug pressure, regardless of the specific resistance mutation. However, while this trend was consistently observed in our tested strains, it may not universally apply to all resistant *A. fumigatus* isolates.

### 3.6. The Correlation Between Resistance and Virulence of A. fumigatus Strains

**Figure 6:**
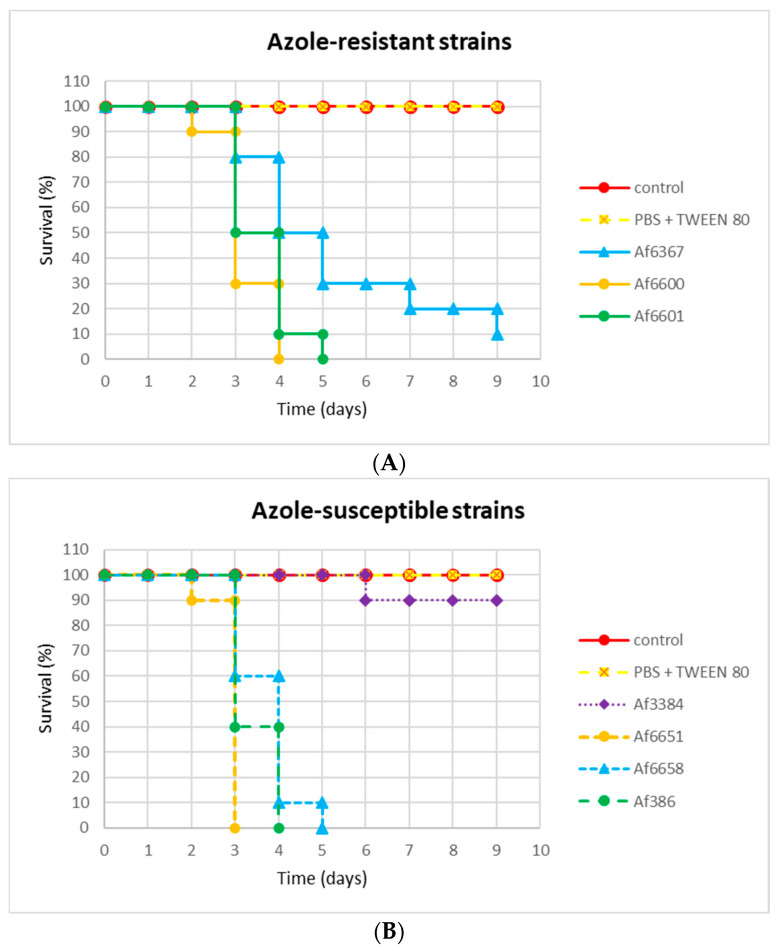
Comparison of the virulence of *A. fumigatus* strains. (**A**) The survival rate of *Galleria mellonella* larvae infected with azole-resistant strains. (**B**) The survival rate of *Galleria mellonella* larvae infected with azole-susceptible strains.

In addition to examining the impact of azole resistance on strain fitness and spread in natural environments, we also investigated the effect of resistance on important aspect of the A. fumigatus strains, the virulence. To compare strain virulence, we employed an invertebrate in vivo model, *Galleria mellonella* (GM), whose immune system resembles human innate immunity. To optimize conditions and ensure objective results, GM larvae from a single generation with the same rearing and storage conditions before and after infection were used. For infection, the same spore concentration (1 × 10^5^ conidia/larva) for each strain was used, determined based on prior testing. The survival of larvae over the course of infection monitoring is presented in Figure 6 using Kaplan–Meier curves.

In most strains (two azole-susceptible and two azole-resistant), infection resulted in 100% mortality on days 4 and 5, respectively. In the case of strain Af6367 with the L98H mutation, the progression of infection was slower compared to other azole-resistant strains, with larvae dying gradually over the entire monitoring period. In contrast, the infection caused by strain Af6651 had a dramatic course, as larvae did not survive beyond 72 h. Strain Af3384 exhibited the lowest virulence potential, with 90% of larvae still alive on day 9 post-infection. These results indicate that azole resistance due to the *cyp51* mutation does not significantly impact the virulence of the strain.

## 4. Discussion

Invasive aspergilloses pose a serious health risk, especially for immunocompromised patients, who frequently experience respiratory complications. The isolates examined in our study originate from such high-risk patients. We analysed clinical isolates of *A. fumigatus* carrying either wild-type or mutated *cyp51A* genes, aiming to compare their resistance, tolerance, and persistence.

According to the agar screening method, the growth on azole-containing agar plates can indicate the presence of specific resistance-conferring mutations. We confirmed this in our strains as well. TR34/L98H is associated with growth on both voriconazole and itraconazole, which we observed for strain Af6367, despite carrying only the L98H mutation. Conversely, growth exclusively on voriconazole suggests the TR46/Y121F/T289A mutation, as confirmed in our testing of strains Af6600 and Af6601 [31].

To gain a comprehensive understanding of susceptibility, we extended our testing beyond azoles to include other antifungal agents commonly used to treat fungal infections. We observed a reduced susceptibility to amphotericin B (AMB) among most isolates, with MIC values exceeding clinical breakpoints. Although resistance to AMB in *Aspergillus* sp. is historically rare [8], elevated MIC values for AMB have been increasingly observed in recent years, possibly due to increased use of AMB as an alternative to azole therapy [32]. Moreover, studies from Brazil and Canada reported high AMB resistance prevalence rates among both clinical and environmental isolates, reaching 27% and 96%, respectively [25,33]. Given these findings, echinocandins may warrant an effective treatment option [25].

Persistence is an emerging phenomenon in fungal pathogenesis that merits increasing attention as it is linked to treatment failure and chronic infections [16]. To assess its clinical relevance, we evaluated the capacity of our isolates to form persistent subpopulations in the context of their antifungal susceptibility. We have shown that persister formation varied among strains: colonies of strains Af386 and Af6658 were macroscopically visible, whereas strain Af3384 required microscopic detection. Previous studies have demonstrated that persister growth rates correlate with drug concentration, meaning that, while persisters can survive high supra-MIC concentrations, they can only proliferate at lower supra-MIC levels [7]. This suggests that, in strains exhibiting persistence, some conidia may remain viable but fail to germinate in the presence of excessive drug concentration. Thus, while *cyp51A* mutations drive resistance, azole-susceptible strains can still persist, complicating treatment outcomes. This is the first description of persister occurrence in strains of *A. fumigatus* from the Czech Republic, causing potential concern in future therapies. Therefore, it is an area meriting further investigation, regarding how persister cells respond to stress conditions macroscopically and at a transcriptomic level, in vitro as well as in vivo.

*Aspergillus* sp. possess a remarkable ability to adapt to diverse stress conditions [34]. This is enabled by a network of stress response pathways that detect and respond to stressors, including antifungal exposure [35]. These pathways enable the fungus to develop tolerance to various stress conditions, including antifungal exposure. Despite some overlap between azole-resistant and azole-susceptible strains in terms of their responses to stress-inducing agents, we observed distinct patterns. Azole-resistant strains exhibited more stable susceptibility profiles, while azole-susceptible strains showed greater variability, suggesting that they may rely more on stress adaptation pathways. The clinical azole-susceptible isolates Af6651, Af6658, and, particularly, Af3384, demonstrated notable deviations, supporting the hypothesis that compensatory signaling plays a key role in these isolates.

This prompted us to question whether azole-resistant strains rely on similar stress-related pathways, such as the calcineurin, TORC1 signaling, or unfolded protein response (UPR), or if they predominantly depend on resistance-conferring mutations. To explore this, we examined the effects of combining azoles with inhibitors of TORC1 and calcineurin, as well as UPR activators.

Attempts to improve susceptibility to voriconazole and itraconazole were unsuccessful, highlighting the robustness of fungal stress response (Table S2). Even azole-susceptible isolates have maintained viability even in the presence of azole inhibitors, indicating that stress pathways can effectively counter external stressors. Significant in vitro effects were only observed when posaconazole, a more potent azole, was used, likely due to its strong inhibition of the Cyp51A enzyme. Structurally, posaconazole contains a long sidechain and an azole ring adjacent to a fluorinated benzene ring, which may enhance its binding affinity. In contrast, itraconazole’s bulkier side chain and chlorine-substituted ring may reduce attachment to the active centrum of Cyp51A, while voriconazole’s simpler structure provides intermediate potency.

Consistent with this structural consideration, we observed enhanced antifungal activity of posaconazole when combined with signaling pathway inhibitors, particularly in the azole-susceptible strain Af6658. Its persistence under antifungal stress supports the idea that susceptible strains may utilize stress signaling, thereby revealing a potential therapeutic vulnerability. Synergistic effects were most evident with FK506 and rapamycin, targeting the calcineurin and TORC1 pathways, suggesting that disruption of these stress adaptive responses sensitizes *A. fumigatus* cells to azoles.

In contrast, azole-resistant strains exhibited enhanced susceptibility only when posaconazole was combined with dithiothreitol (DTT). Unlike the targeted action of FK506 or rapamycin, DTT acts non-specifically, reducing disulfide bonds in numerous proteins, which affects protein folding, redox homeostasis, and cell wall integrity. It can also reduce oxidized glutathione (GSSG), contributing to reductive stress. Therefore, the observed increase in posaconazole activity is likely due to widespread cellular disruption rather than inhibition of a specific signaling pathway.

In conclusion, our findings highlight the central role of stress adaptation pathways in fungal survival and point to the potential of combinational antifungal strategies targeting multiple cellular processes. Although compounds like FK506, rapamycin, and DTT are not antifungals per se, their modulatory effects offer insights into fungal biology and underscore the need for deeper investigation into the interplay of resistance, tolerance, and persistence in *A. fumigatus*.

Recent research suggests that resistance development may be influenced by defects in the DNA mismatch repair (MMR) system, which could predispose fungi to resistance against both currently used and newly developed antifungals. However, mutations occurring at other loci may not always be beneficial and can sometimes lead to decreased competitive fitness in a stable environment [36]. While mixed-species infections are common, we evaluated the fitness cost of our isolates by one-to-one competition assays [20]. Our findings revealed that voriconazole-susceptible strains (Af386, Af6658) gradually outcompeted voriconazole-resistant strains (Af6367, Af6600), indicating a fitness cost associated with azole resistance in strains containing the L98H mutation as well as strains containing the TR46/Y121F/T289A mutation. This is not unprecedented, as similar observations were made in Chen et al., 2024 [20]. However, previous studies have refuted a direct relationship between *cyp51A* mutations and fitness cost [20,37], suggesting that reduced competitiveness is more likely linked to other variable mutations carried by resistant strains. It would also be of value to test fitness costs in the presence of stress-inducing agents in future studies. However, this area remains challenging as clearly distinctive susceptibility profiles for tested strains were not detected (Figure S2 and S3 compared to Figure S1 for voriconazole).

On the other hand, the effect of azole resistance on the virulence of the strains was not evident in our testing. To compare the virulence potential of the strains, *Galleria mellonella* larvae were used. Both the most and the least virulent strains were azole-susceptible, while the survival rate of larvae infected with azole-resistant strains fell within the average range. Our results suggest that virulence is primarily a strain-specific feature and is not influenced by *cyp51*-mediated resistance.

## 5. Conclusions

Our study of clinical *Aspergillus fumigatus* isolates from the Czech Republic highlights that stress adaptation pathways play a critical role in the survival and antifungal response of *Aspergillus fumigatus*, regardless of the strain’s resistance profile. While azole-resistant isolates rely primarily on *cyp51A* mutations, our findings suggest that both resistant and susceptible strains retain functional stress response systems that help them withstand antifungal pressure. Despite being classified as azole-susceptible, many strains displayed a strong ability to form persisters, maximizing population survival under stress. This indicates that stress signaling can compensate for the absence of resistance-conferring mutations, causing potential concern for future therapies. Nevertheless, only a very potent azole (posaconazole and beyond) could shed light on adaptation mechanisms as discussed above.

Though the azole-resistance may come at the cost of fitness, the strains have comparable virulence strength. Overall, our findings underscore the multifaceted nature of antifungal resistance and tolerance in *A. fumigatus* and suggest that targeting stress adaptation mechanisms could be a promising strategy to improve treatment outcomes. Therefore, future research should prioritize strategies to counteract both resistance and persistence (including creation of standardised protocols assessing persister formation), ensuring more effective antifungal interventions.

## Figures and Tables

**Table 1:** Characterization of *A. fumigatus* strains.

Clinical Isolates							
Strain Code	Isolation			Patient			Mutations
	Substrate	Year	Locality	Diagnosis/Health Issues	Age	Gender	*Cyp51a* Gene
CCF 6600	endotracheal aspirate	2021	Hospital with polyclinic Karviná-Ráj, Czech Republic	COVID-19 bronchopneumonia	76	woman	TR46/Y121F/T289A
CCF 6601	sputum	2021	Internal department, City hospital Ostrava, Czech Republic	COVID-19 bronchopneumonia	67	woman	TR46/Y121F/T289A
CCF 6367	sputum	2018	Pneumology department, Regional hospital Kladno, Czech Republic	cough	80	man	L98H
CCF 3384	sputum	2001	Hradec Králové, Czech Republic	-	57	woman	none
CCF 6651	sputum	2019	Praha, Czech Republic	after lung transplantation	69	man	none
CCF 6658	sputum	2020	Ostrava, Czech Republic	acute suppurative otitis	66	woman	none
**Environmental isolates**							
**Strain Code**	**substrate**						
CCM F-386	tropical soil						

**Table 2:** Antifungal susceptibility of *A. fumigatus* strains based on MIC values. MIC values were determined and compared with MIC breakpoints (MIC B) and clinical breakpoints (CB) as defined by The European Committee on Antimicrobial Susceptibility Testing (EUCAST, 2024). Susceptibility classifications: S-susceptible, R-resistant, ND-not defined. MIC values were obtained for voriconazole (VRC), itraconazole (ITC), posaconazole (PSC), and amphotericin B (AMB). For caspofungin (CAS), anidulafungin (ANI), and micafungin (MIK), MIC80 values were determined.

	MIC B/CB (mg/L)		*A. fumigatus* Isolates						
	S≤	R>	Af6367	Af6600	Af6601	Af386	Af3384	Af6651	Af6658
VRC	1	1	2.5	>10	>10	0.5	1	1	0.5
ITC	1	1	2.5 **	5	5	0.5	1	1	0.5
PSC	0.125	0.25	0.25	0.25	0.1	0.05	0.1	0.05	0.05
AMB	1	1	5	5	1	5	5	5	5
CAS *	ND	ND	0.5	0.5	0.1	0.5	0.1	0.5	0.3
MIK *	ND	ND	0.005	0.005	0.005	0.005	0.01	0.005	0.005
ANI *	ND	ND	0.01	0.01	0.005	0.01	0.01	0.005	0.005

* MIC80; ** MIC70.

**Table 3:** Susceptibility of *A. fumigatus* to various stress-inducing agents. Compounds are categorised based on their primary cellular targets or mechanisms of action. Results are expressed as MIC80 values (mean ± SD), except for rapamycin (* MIC60 values) and Af3384 (** MIC 40 value). Abbreviations: DTT—dithiothreitol, SDS—sodium dodecyl sulfate, 4 NQO—4-nitroquinoline-1-oxide.

Type of Stress	Stress-Inducing Agents		Af6367	Af6600	Af6601	Af386	Af3384	Af6651	Af6658
cell wall stress	Congo red	mM	1.8 ± 0.9	3.5 ± 0.7	1.0 ± 0.2	7.1 ± 4.2	3.2 ± 0.5	5.0 ± 1.2	1.0 ± 0.2
osmotic stress	NaCl	M	1.6 ± 0.2	1.6 ± 0.30	1.5 ± 0.05	1.6 ± 0.2	1.7 ± 0.25	0.95 ± 0.25	1.5 ± 0.05
	LiCl	mM	51 ± 1.7	43 ± 1.4	48.5 ± 2.0	90 ± 8.5	107 ± 5.0	44 ± 5.7	70 ± 2.0
oxidative stress	H_2_O_2_	mM	4.6 ± 0.06	4.8 ± 0.7	4.5 ± 0.07	4.4 ± 0.09	6.7 ± 0.2	3.6 ± 0.7	6.8 ± 0.03
	menadione	mg/L	0.04 ± 0.01	0.07 ± 0.03	0.07 ± 0.04	0.04 ± 0.005	0.04 ± 0.005	0.02 ± 0.003	0.02 ± 0.001
signal pathways interference	rapamycin *	mg/L	0.005 ± 0.001	0.05 ± 0.01	0.005 ± 0.001	0.01 ± 0.002	0.04 ± 0.01 **	0.1 ± 0.02	0.01 ± 0.003
	FK 506	mg/L	0.07 ± 0.03	0.05 ± 0.004	0.05 ± 0.003	0.05 ± 0.004	0.5 ± 0.01	0.2 ± 0.1	0.05 ± 0.007
	cyclosporine A	mg/L	2.2 ± 0.4	0.8 ± 0.3	0.8 ± 0.3	5 ± 0.8	10 ± 2.5	5.5 ± 0.6	3.3 ± 0.4
endoplasmic reticulum stress	DTT	mM	43 ± 6.5	47 ± 3.5	41 ± 2.9	44 ± 4.0	43 ± 5.9	41 ± 9.5	54 ± 13.9
	brefeldin A	mg/L	9.5 ± 5.5	13 ± 3.9	6.9 ± 2.6	15.8 ± 5.1	25 ± 0.5	31.5 ± 20	7.9 ± 2.6
	tunicamycin	mg/L	48 ± 3.5	50 ± 2.5	>100	100 ± 2	50 ± 5	100 ± 5	50 ± 1.5
others	4 NQO	mg/L	2.5 ± 0.07	0.70 ± 0.1	1.0 ± 0.04	0.70 ± 0.15	0.95 ± 0.15	1.8 ± 0.1	1.7 ± 0.1
	benomyl	mg/L	>100	>100	>100	0.7 ± 0.25	2.3 ± 0.7	4.0 ± 0.8	0.8 ± 0.05
	SDS	%	0.04 ± 0.001	0.03 ± 0.01	0.04 ± 0.001	0.03 ± 0.01	0.04 ± 0.0007	0.02 ± 0.002	0.02 ± 0.005

**Table 4:** Comparison of azole-susceptible and azole-resistant strains based on MIC80 values for individual stress-inducing compounds (* MIC60, (MIC40 for Af3384) values comparison for rapamycin). Results are presented as mean ± SD. Statistical significance was assessed by Student’s *t*-test with a *p*-value threshold of 0.05.

Stress-Inducing Agent		Resistant Strains	Susceptible Strains	*p*-Value
SDS	%	0.04 ± 0.01	0.02 ± 0.01	0.001
cyclosporine A	mg/L	1.16 ± 0.69	6.23 ± 3.10	0.010
menadione	mg/L	0.07 ± 0.03	0.02 ± 0.01	0.016
FK506	mg/L	0.06 ± 0.02	0.27 ± 0.22	0.042
brefeldin A	mg/L	9.56 ± 4.54	21.5 ± 14.7	0.043
H_2_O_2_	mM	4.61 ± 0.41	5.71 ± 1.61	0.079
LiCl	mM	48.0 ± 3.87	69.1 ± 28.4	0.097
NaCl	M	1.55 ± 0.20	1.36 ± 0.39	0.231
Congo red	mM	2.03 ± 1.18	3.05 ± 1.80	0.260
rapamycin *	mg/L	0.02 ± 0.02	0.06 ± 0.05	0.278
DTT	mM	43.9 ± 5.13	45.3 ± 10.7	0.709
tunicamycin	mg/L	65.8 ± 26.5	70.0 ± 27.4	0.805
4NQO	mg/L	1.27 ± 0.75	1.33 ± 0.43	0.835

## Data Availability

The original contributions presented in this study are included in the article/Supplementary Material. Further inquiries can be directed to the corresponding authors.

## Supplementary Materials

The following supporting information can be downloaded at: https://www.mdpi.com/article/10.3390/jof11060428/s1, Table S1: The range of concentrations of antifungal agents and stress-inducing agents for susceptibility testing; Table S2: Evaluation of the potential synergistic effect of azoles and signaling pathway modulators based on a comparison of fungal growth percentage (mean ± SD) in the presence of both compounds versus each compound individually; Table S3: The number of persisters formed by *A. fumigatus* strains in the presence of azoles at supra-MIC concentrations applied to a paper disc; Figure S1: Growth dependence (%) of each strain on azole concentration; Figure S2: Growth dependence (%) of each strain on stress-inducing agent concentration: dithiothreitol, brefeldin A, tunicamycin; Figure S3: Growth dependence (%) of each strain on stress-inducing agent concentration: rapamycin, FK 506, cyclosporine A.
