# Supplementary material for: Adaptation Under Pressure: Resistance and Stress Response Interplay in Clinical Aspergillus fumigatus Isolates
Source: J Fungi (Basel). 2025 Jun 2;11(6):428. doi: 10.3390/jof11060428 (PMC12194776; doi:10.3390/jof11060428)
Supplement: Supplementary file 1 [file jof-11-00428-s001.zip › jof-3597658-supplementary.pdf]

Supplementary materials

Table S1. The range of concentrations of antifungal agents and stress-inducing agents for susceptibility testing. (SDS - sodium dodecyl sulfate, 4 NQO - 4-nitroquinoline-1-oxide)

**Antifungals**		range of concentrations (mg/L)
Azoles	Voriconazole	0.01 - 10
	Itraconazole	0.01 - 10
	Posaconazole	0.01 - 1
Polyenes	Amphotericin B	0.5 - 10
Echinocandins	Caspofungin	0.01 - 1
	Micafungin	0.001 - 1
	Anidulafungin	0.001 - 1

**Stress-inducing agents**		range of concentrations
cell wall stress	Congo red	0.1 - 10 mM
osmotic stress	NaCl	0.1 - 2 M
	LiCl	10 - 150 mM
oxidative stress	H_2O_2	1 - 10 mM
	menadione	0.01 - 1 mg/L
signal pathways interference	rapamycin	0.001 - 10 mg/L
	FK 506	0.001 - 1 mg/L
	cyclosporine A	0.01 - 10 mg/L
endoplasmic reticulum stress	dithiothreitol	10 - 50 mM
	brefeldin A	0.1 - 50 mg/L
	tunicamycin	1 - 100 mg/L
others	4 NQO	0.1 - 5 mg/L
	benomyl	0.1 - 100 mg/L
	SDS	0.005 - 0.5 %

Table S2. Evaluation of the potential synergistic effect of azoles and signaling pathway modulators based on a comparison of fungal growth percentage (mean ± SD) in the presence of both compounds versus each compound individually. Abbreviations: VRC-voriconazole, ITC-itraconazole, PSC-posaconazole, DTT-dithiothreitol, TUN-tunicamycin, BREF A-brefeldin A, RAP-rapamycin, CYCLO A-cyclosporine A, SD- standard deviation.

**Af6600**				**Af6658**			
compound	concentration (mg/L) (*mM)	% of growth ± SD	potential synergistic effect	compound	concentration (mg/L) (*mM)	% of growth ± SD	potential synergistic effect
voriconazole	10	55.7 ± 5.40	-	voriconazole	0.1	61.8 ± 1.69	-
itraconazole	0.25	52.0 ± 1.16	-	itraconazole	0.02	67.5 ± 6.02	-
posaconazole	0.25	48.0 ± 2.87	-	posaconazole	0.02	63.8 ± 3.10	-
dithiothreitol*	25	38.9 ± 1.08	-	dithiothreitol*	25	47.7 ± 3.31	-
tunicamycin	10	50.3 ± 3.80	-	tunicamycin	10	52.2 ± 0.45	-
brefeldin A	1	52.1 ± 0.35	-	brefeldin A	1	54.8 ± 3.93	-
FK 506	0.005	50.0 ± 6.70	-	FK 506	0.003	63.6 ± 7.21	-
rapamycin	0.005	54.8 ± 11.9	-	rapamycin	0.005	44.6 ± 6.98	-
cyclosporine A	0.1	50.6 ± 5.86	-	cyclosporine A	0.1	59.1 ± 2.16	-
VRC + DTT*	10 + 25	51.0 ± 5.37	no	VRC + DTT*	0.1 + 25	50.1 ± 1.23	no
VRC + TUN	10 + 10	52.3 ± 1.82	no	VRC + TUN	0.1 + 10	53.8 ± 1.09	no
VRC + BREF A	10 + 1	43.6 ± 1.05	no	VRC + BREF A	0.1 + 1	43.0 ± 0.76	no
VRC + FK 506	10 + 0.005	43.9 ± 3.22	no	VRC + FK 506	0.1 + 0.003	50.8 ± 3.91	no
VRC + RAP	10 + 0.005	45.0 ± 3.05	no	VRC + RAP	0.1 + 0.005	45.2 ± 1.10	no
VRC + CYCLO A	10 + 0.1	46.9 ± 0.98	no	VRC + CYCLO A	0.1 + 0.1	53.8 ± 2.51	no
ITC + DTT*	0.25 + 25	37.7 ± 3.94	no	ITC + DTT*	0.02 + 25	43.7 ± 7.52	no
ITC + TUN	0.25 + 10	50.7 ± 2.39	no	ITC + TUN	0.02 + 10	47.4 ± 4.36	no
ITC + BREF A	0.25 + 1	39.1 ± 4.76	no	ITC + BREF A	0.02 + 1	41.6 ± 4.08	no
ITC + FK 506	0.25 + 0.005	45.0 ± 3.05	no	ITC + FK 506	0.02 + 0.003	53.4 ± 8.79	no
ITC + RAP	0.25 + 0.005	38.12 ± 10.1	no	ITC + RAP	0.02 + 0.005	42.6 ± 8.27	no
ITC + CYCLO A	0.25 + 0.1	47.5 ± 4.88	no	ITC + CYCLO A	0.02 + 0.1	51.8 ± 3.88	no
PSC + DTT*	0.25 + 25	0 ± 0	yes	PSC + DTT*	0.02 + 25	16.1 ± 1.92	yes
PSC + TUN	0.25 + 10	43.5 ± 2.12	no	PSC + TUN	0.02 + 10	44.2 ± 1.11	no
PSC + BREF A	0.25 + 1	45.2 ± 2.55	no	PSC + BREF A	0.02 + 1	35.2 ± 1.67	yes
PSC + FK 506	0.25 + 0.005	40.3 ± 2.35	no	PSC + FK 506	0.02 + 0.003	23.1 ± 1.26	yes
PSC + RAP	0.25 + 0.005	42.3 ± 2.43	no	PSC + RAP	0.02 + 0.005	16.3 ± 3.08	yes
PSC + CYCLO A	0.25 + 0.1	34.5 ± 4.89	no	PSC + CYCLO A	0.02 + 0.1	52.8 ± 2.51	no

Table S3. The number of persisters formed by *A. fumigatus* strains in the presence of azoles at supra-MIC concentrations applied to a paper disc. (voriconazole (VRC), itraconazole (ITC), posaconazole (PSC), "-" not assessed)

azole	concentration (mg/L)	Af6367	Af6600	Af6601	Af386	Af3384	Af6651	Af6658
VRC	2	-	-	-	0	0	0	17
	5	0	-	-	16	0	0	27
	10	0	-	-	52	8	0	14
ITC	4	-	-	-	0	0	0	0
	10	-	0	0	0	0	0	0
PSC	0.5	0	0	0	0	0	0	0
	5	0	0	0	0	0	0	18

Figure S1. Growth dependence (%) of each strain on azole concentration: voriconazole (VRC), itraconazole (ITC), posaconazole (PSC), in mg/L. Experiments were performed 3 times, with each point representing the mean ± SD.

Figure S2. Growth dependence (%) of each strain on stress-inducing agent concentration: dithiothreitol (DTT, mM), brefeldin A, tunicamycin (both in mg/L). Experiments were performed 3 times, with each point representing the mean ± SD.

Figure S3. Growth dependence (%) of each strain on stress-inducing agent concentration: rapamycin, FK 506, cyclosporine A (each in mg/L). Experiments were performed 3 times, with each point representing the mean ± SD.

Figure S1

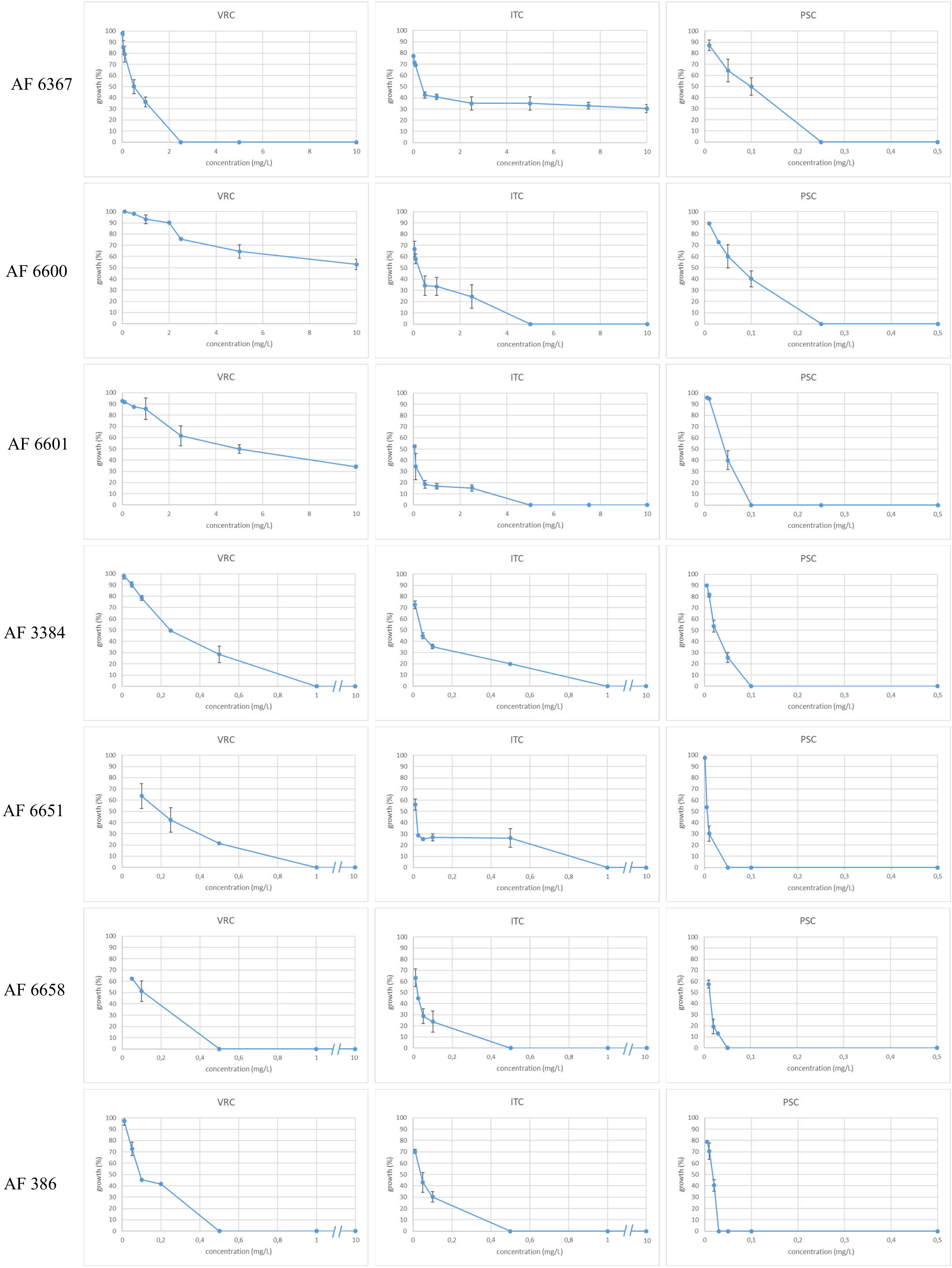

AF 6367
AF 6600
AF 6601
AF 3384
AF 6651
AF 6658
AF 386
VRC
ITC
PSC
growth (%)
concentration (mg/L)

Figure S2

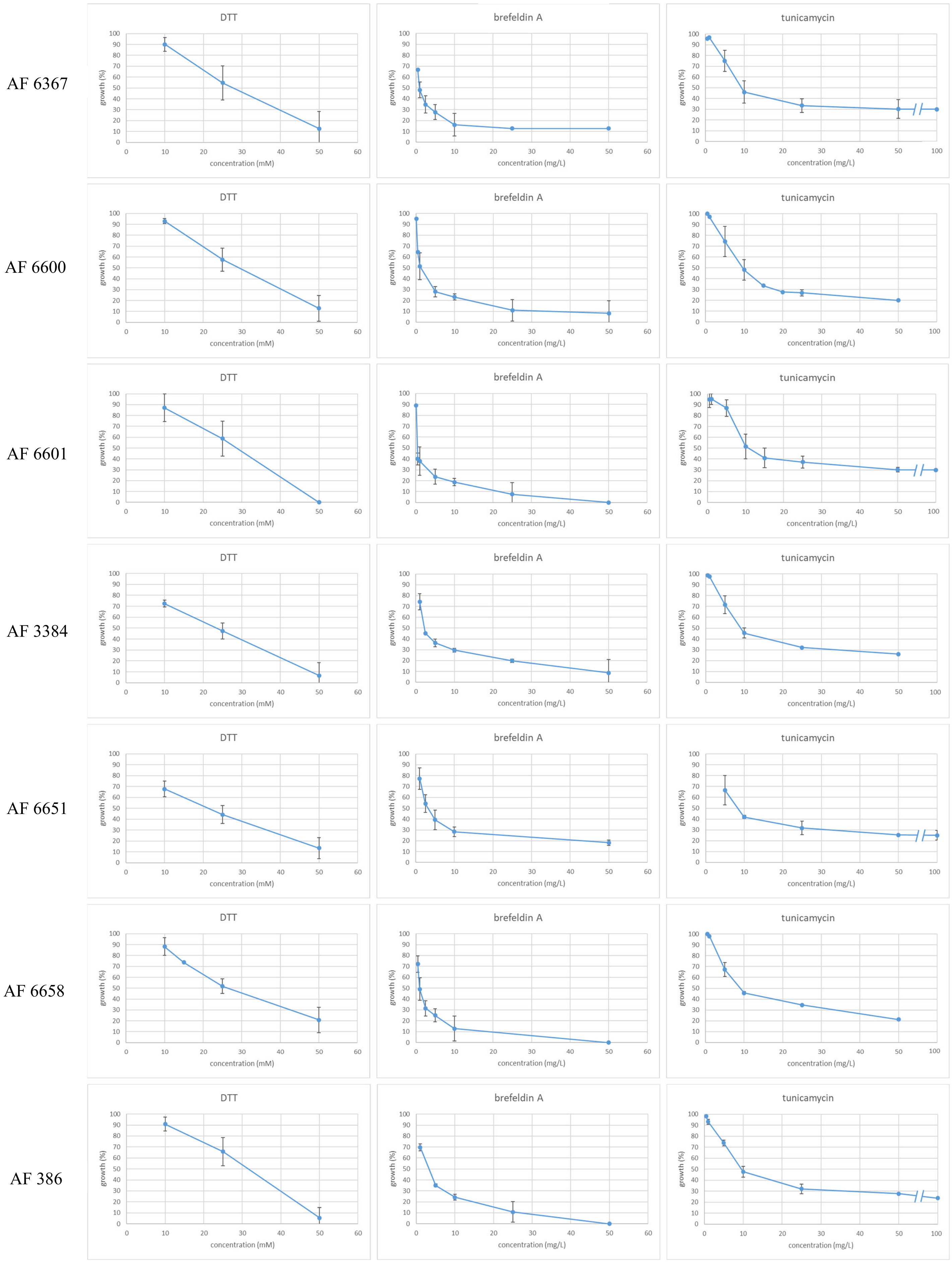
AF 6367
AF 6600
AF 6601
AF 3384
AF 6651
AF 6658
AF 386
DTT
brefeldin A
tunicamycin
growth (%)
concentration (mM)
concentration (mg/L)

Figure S3

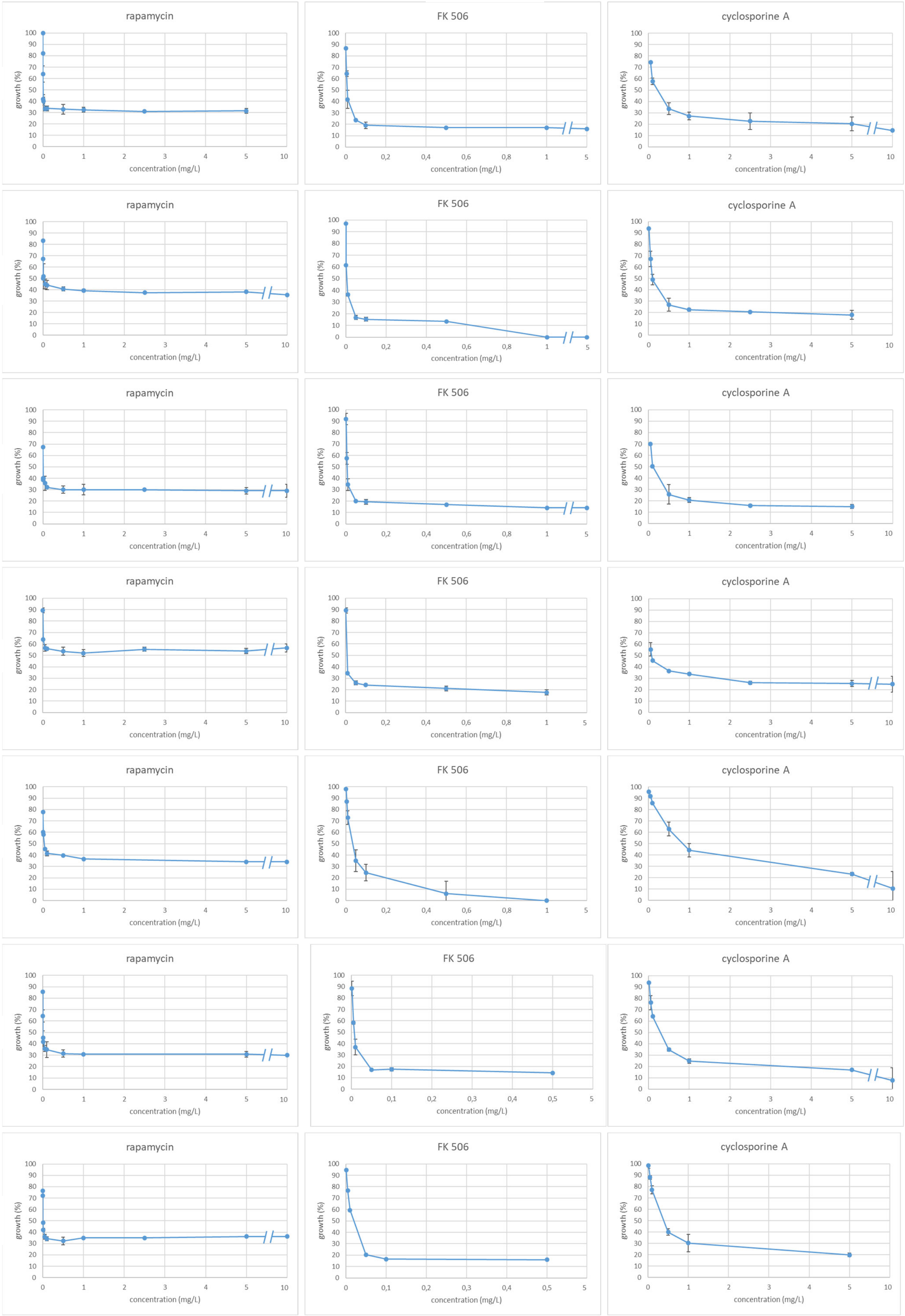
AF 6367
AF 6600
AF 6601
AF 3384
AF 6651
AF 6658
AF 386
rapamycin
FK 506
cyclosporine A
growth (%)
concentration (mg/L)
